# Ecophysiological responses to heat waves in the marine intertidal zone

**DOI:** 10.1242/jeb.246503

**Published:** 2025-01-16

**Authors:** Jonathon H. Stillman, Adrienne B. Amri, Joe M. Holdreith, Alexis Hooper, Rafael V. Leon, Liliana R. Pruett, Buck M. Bukaty

**Affiliations:** ^1^ Department of Biology, San Francisco State University, San Francisco, CA 94132, USA; ^2^ Department of Integrative Biology, University of California Berkeley, Berkeley, CA 94709, USA

**Keywords:** Plasticity, Adaptation, Multiple stressor, Disease, Microhabitat, Stress, Physiology, Behavior, Energetics, Cellular stress response

## Abstract

One notable consequence of climate change is an increase in the frequency, scale and severity of heat waves. Heat waves in terrestrial habitats (atmospheric heat waves, AHW) and marine habitats (marine heat waves, MHW) have received considerable attention as environmental forces that impact organisms, populations and whole ecosystems. Only one ecosystem, the intertidal zone, experiences both MHWs and AHWs. In this Review, we outline the range of responses that intertidal zone organisms exhibit in response to heat waves. We begin by examining the drivers of thermal maxima in intertidal zone ecosystems. We develop a simple model of intertidal zone daily maximum temperatures based on publicly available tide and solar radiation models, and compare it with logged, under-rock temperature data at an intertidal site. We then summarize experimental and ecological studies of how intertidal zone ecosystems and organisms respond to heat waves across dimensions of biotic response. Additional attention is paid to the impacts of extreme heat on cellular physiology, including oxidative stress responses to thermally induced mitochondrial overdrive and dysfunction. We examine the energetic consequences of these mechanisms and how they shift organismal traits, including growth, reproduction and immune function. We conclude by considering important future directions for improving studies of the impacts of heat waves on intertidal zone organisms.

## Teaser

**Summary:** Heat waves are intensifying with climate change. We review recent literature that highlights causes and physiological impacts of heat waves on intertidal zone organisms, including mortality, plasticity and adaptation.

## Introduction

### Atmospheric and marine heat waves

A central consequence of global warming is an increase in persistent extreme high temperatures, or heat waves (Domeisen et al., 2023; Perkins-Kirkpatrick et al., 2024). When sea surface temperatures (SSTs) exceed the 90th-percentile temperature for at least 5 consecutive days, the thermal variation is considered a marine heat wave (MHW; see Glossary; Hobday et al., 2018; Oliver et al., 2021). MHWs increased by 34% in frequency and 17% in duration between 1925 and 2016 (Oliver et al., 2018) and are occurring over a 24% greater area of the Earth's oceans (Hobday et al., 2018). Abnormally hot weather (>95th percentile) over terrestrial regions lasting for at least 2 consecutive days is defined as an atmospheric heat wave (AHW; see Glossary) (Barriopedro et al., 2023; IPCC, 2023). AHWs across the USA have increased steadily, from an average of two per year in the 1960s to six per year during the 2010s and 2020s and currently last a day longer than the average AHW in the 1960s (EPA, 2024). Under current climate models (IPCC, 2023), the frequency, geographic extent and intensity of AHWs are expected to continue to increase, posing major risks to biodiversity and ecosystem function (Perkins-Kirkpatrick and Lewis, 2020). In some coastal regions, linkages between MHWs and AHWs are likely. For example, warm phases of ENSO that co-occur with Indian Ocean MHWs have increased the occurrence of AHWs in India, China, Europe and Northeast Asia (Barriopedro et al., 2023).

**Glossary**

**Atmospheric heat wave (AHW)**

A period of extremely warm weather of at least the 95th percentile of temperatures for that area that persists for at least 2 consecutive days.

**Cellular stress response (CSR)**

A coordinated set of responses involving gene expression and protein synthesis and modification that shifts the cellular physiology to cope with dangerous situations that can result in problems with cellular and physiological homeostasis.

**Critical thermal maximum (CT**_**max**_**)**

The upper temperature at which failure of coordinated organismal function associated with locomotion or righting response occurs.

**DNA methylation**

A form of epigenetic change caused by the addition of a methyl group to the DNA backbone that results in an alteration in the expression of genes.

**Heat hardening**

A physiological response to acute exposure to high temperature that increases tolerance to increased temperature for an extended period of time.

**Heat shock proteins (HSPs)**

A class of chaperone proteins that are regulated by stress responsive pathways.

**Heat softening**

A physiological response to acute exposure to high temperature that decreases tolerance to subsequent exposure to increased temperature.

**LT50**

The temperature that causes 50% lethality in a population under a specific temporal framework.

**Marine heat wave (MHW)**

A period of extremely warm water above the 90th percentile of temperatures for that area that persists for at least 5 consecutive days.

**Reactive oxygen species (ROS)**

A group of highly reactive oxygen-containing compounds with unpaired electrons, produced during mitochondrial dysfunction.

**Sea surface temperature (SST)**

The temperature of the top few millimeters of ocean water.

**Transgenerational plasticity (TGP)**

A change in the cellular composition of gametes due to the environment during oogenesis and spermatogenesis that causes phenotypic shifts in the next generation. TGP can be heritable (e.g. epigenetic modification) or non-heritable (e.g. shifts in maternally provisioned proteins in the oocyte).

**Vertical zones**

Elevational bands containing different species assemblages in intertidal habitats which are set by physiological tolerance to aerial exposure and ecological interactions.

### Heat waves in the intertidal zone

**Fig. 1.**
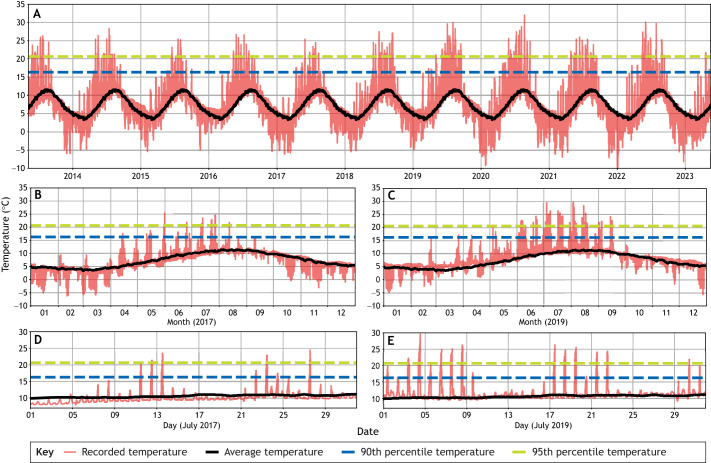
**Temperature profiles in the intertidal zone at Kachemak Bay, AK, USA, during the period 2012–2023.** (A) One decade of data showing temperature data at 30 min intervals (red), daily average temperature (black), 90th percentile of daily maximum temperature (blue dashed line) and 95th percentile of daily maximum temperature (green dashed line) for all calendar days across the entire dataset. (B) One year of data (2017) during a relatively cool year. (C) One year of data (2019) during a relatively hot year. (D) One month of data (July 2017) during the hottest part of a relatively cool year. (E) One month of data (July 2019) during the hottest part of a relatively hot year. Data are from USGS Alaska Science Center (US Geological Survey data release, v.4.0; https://doi.org/10.5066/F7WH2N3T).

Intertidal zone temperatures are highly variable, with daily variation during cycles of tidal immersion and emersion superimposed on seasonal variation in average air and water temperatures, and – in upwelling zones – seasonal upwelling of colder water (Fig. 1). The intertidal zone also presents one of nature's steepest thermal gradients. As a result, the organisms of the intertidal zone have been excellent models for the study of thermal adaptation and response to global warming (Blewett et al., 2022; Dong, 2023; Dong et al., 2022; Somero, 2002). The intertidal zone is the largest environment in which organisms experience MHWs and AHWs, as it is the habitat at the interface of ocean and land and is exposed to changes in marine and atmospheric conditions. Thus, intertidal zone organisms are at risk of experiencing a combination of extreme water and air temperatures.

Intertidal zone organisms are stratified into different vertical zones (see Glossary) based on tolerance to the abiotic stresses of emersion (e.g. temperature and desiccation) and biotic interactions (e.g. competition, predation) (Benjamin et al., 2024; Connell, 1961; Stillman and Somero, 2000; Ziegler et al., 2023). The degree to which AHW and MHW conditions are experienced depends on the vertical distribution of intertidal zone organisms. Organisms living above the middle intertidal zone experience emersion daily and can spend ≥50% of their lives out of water (Stillman and Somero, 1996). Those organisms are much more likely to experience AHWs simply as a consequence of the duration of time that they are exposed to air. Conversely, intertidal organisms living low on the shore, where emersion only occurs during spring tides (maximal tidal amplitude), are much more likely to experience MHWs as they spend the majority of their lives immersed. When conditions are such that MHWs and AHWs occur simultaneously, intertidal zone organisms will experience extreme temperatures while immersed at high tide and emersed at low tide, though the levels of heat in water and air are likely to differ. The maximum vertical position of intertidal zone organisms is typically set by physiological tolerance limits (Stillman and Somero, 2000), and any further increase in abiotic stressors, such as MHWs and AHWs, is likely to have detrimental physiological consequences (Stillman, 2019). In addition to exposure to increased frequency, duration and magnitude of thermal extremes, intertidal zone organisms routinely experience variability in other environmental drivers (e.g. pH, dissolved oxygen), making the intertidal zone a multifarious or multi-stressor environment (Blewett et al., 2022; Gunderson et al., 2016). Among these drivers, in a multi-stressor context, temperature is typically the strongest environmental factor regulating overall organismal function (Paganini et al., 2014), except in cases of extreme hypoxia resulting from upwelling of anoxic waters to coastal habitats, which causes mass mortality (Grantham et al., 2004). Intertidal organisms are adapted to life in a dynamic environment with intersecting natural lunar (tidal) and solar cycles, and they adjust their physiological and behavioral responses (e.g. phenotypic plasticity) in a rhythmic synchrony with those environmental cycles (e.g. Connor and Gracey, 2020). The extent to which AHWs and MHWs influence the endogenous rhythmicity of physiology in intertidal zone organisms is yet to be determined.

**Fig. 2.**
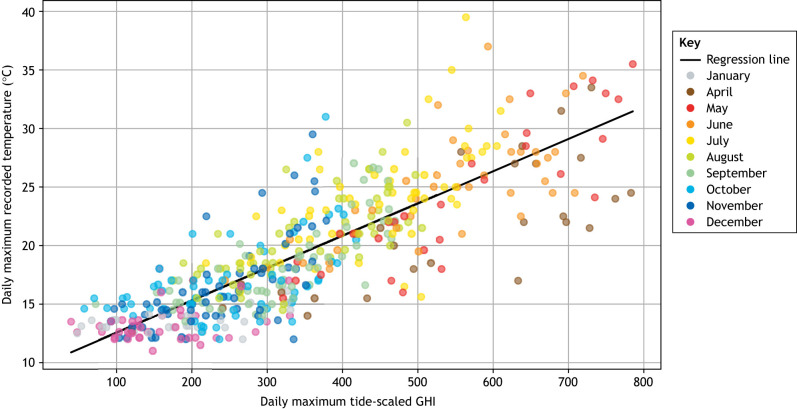
**Comparison of calculated solar energy reaching the mid-intertidal zone during low tide with daily thermal maximum recorded underneath stones in the mid-intertidal zone at Fort Ross, CA, USA.** Data are from Gunderson et al. (2019). GHI, global horizontal irradiance. The linear regression fit equation is *y*=9.79+0.0276*x*, *P*=8.3e−117, *r*^2^=0.686. See Supplementary Materials and Methods for a full description of the method.

While the temperatures that intertidal zone organisms experience are influenced by air and water temperatures, a key determinant of organismal temperature is the absorption of solar radiation (Helmuth, 2002), which is a function of organismal size, color and solar exposure angle (Helmuth et al., 2010). The amount of solar energy that reaches the intertidal zone during low tide has been shown to impact intertidal zone organisms and shift intertidal zone ecosystems (Hesketh and Harley, 2023). Thus, how AHW and MHW conditions influence intertidal zone ecosystems will also depend on solar radiation absorption in the intertidal zone, which varies by time of day, season and cloud albedo. The importance of solar radiation as a determining factor of maximum intertidal zone body temperatures has been demonstrated by a model that we have built and that intersects spatially high-resolution modeled sun exposure data from the National Solar Radiation Database (NSRDB) Physical Solar Model 3 (PSM3) with tidal height predictions from the National Oceanic and Atmospheric Administration (NOAA). We applied this model to an intertidal zone site at Fort Ross, CA, USA where high-resolution temperature data have been recorded underneath mid-intertidal zone rocks (Gunderson et al., 2019). Incorporating the assumption that sun exposure during periods of high tide will not cause thermal increases in submerged portions of the intertidal zone, we transformed the timestamped solar radiation data (specifically, global horizontal irradiance or GHI) by scaling the solar radiation by the inverse tidal height (for detailed methods of the model, please see the Supplementary Materials and Methods). The resulting tide-scaled GHI roughly represents the solar radiation directly absorbed by the intertidal zone substratum and accounted for 69% of the variation in daily thermal maxima at the Fort Ross site (Fig. 2). This result suggests that other factors, including wind and environmental temperatures during AHW and MHW conditions, contribute to just 31% of the variation in daily thermal maxima under intertidal zone rocks at this location. At Fort Ross, the time of year during which low tides are closest to midday is in the early spring, and not surprisingly maximal temperatures are observed at that time of year (Fig. 2). As solar radiation is not expected to shift during the current period of climate change (Foukal et al., 2006), AHW increases in maximum air temperature may have a muted effect on maximum temperatures experienced in intertidal zone environments as compared with terrestrial environments.

Despite the wealth of studies on the thermal physiology of intertidal zone organisms that focus on adaptation to the intertidal zone thermal environment, there have been fewer studies that focus specifically on understanding physiological responses of marine intertidal organisms to thermal extremes associated with MHW and AHW conditions. In this Review, we attempt to synthesize and summarize recent studies on the impacts of heat waves on intertidal zone organisms. We specifically set out to provide a synopsis of studies that examined shifts in the thermal environment of intertidal zone organisms that constituted a heat wave – those studies being a specific type of investigation of physiological responses to elevated temperature and thermal stress. To better understand the importance of the frequency and magnitude of heat waves in causing lethality, we review examples of heat waves that have been intense enough to cause mass mortality in intertidal zone communities. Next, we consider evidence for heat waves to accelerate adaptive responses in populations through selection, genome rearrangement and trans-generational plasticity effects. Subsequently, we consider within-generation plasticity, highlighting responses to heat waves in which organisms become increasingly heat tolerant (i.e. heat hardened) or decreasingly heat tolerant (i.e. heat softened) following exposure to heat wave conditions. Mechanisms involved with heat hardening and softening (see Glossary) including mitochondrial function (or dysfunction), the cellular stress response (and its limits), and oxidative stress responses and accumulation of oxidative damage are presented using examples specific to intertidal zone animals in a heat wave context. Finally, we consider the energetic consequences of responses to heat waves by intertidal zone animals and their effect on organismal traits such as reproduction, behavior, immune and host–parasite responses.

## Population consequences of heat waves in the intertidal zone

### Mass mortality

In general, mass mortality is when the majority of the organisms within a population die within a short amount of time. That time frame depends on the type and intensity of the heat wave and could range from weeks to months during MHWs or occur within a single low-tide period (6–8 h) during AHWs that reach lethal temperatures. For example, the keystone predator sun star *Pycnopodia helianthoides* experienced a reduction of 80–100% of its historical population size over its entire 3000 km range, with MHW conditions and sea star wasting disease being the causative factors (Harvell et al., 2019). Mass mortality in response to AHWs has been observed in intertidal clams from the South China sea that experienced air temperatures of >50°C during low tide (He et al., 2022a,b) and in kelp exposed to MHWs in New Zealand (Thomsen et al., 2019). During the historically high extreme AHW during the June 2021 Pacific Northwest heat wave, in which maximal temperatures occurred during midday low tides in the Salish Sea for several consecutive days, mass mortality was observed in areas also exposed to direct solar radiation (Hesketh and Harley, 2023). The extreme heat wave resulted in the loss of 74% of oysters and 81% of barnacles (Raymond et al., 2022). Despite the historically high temperatures during that AHW, air temperatures alone were insufficient to cause mass mortality (Hesketh and Harley, 2023). Biomimetic temperature dataloggers in mussel beds during a mass mortality event showed that mussel body temperature was consistently significantly warmer than air temperature and warmer than the bare rocks or the rocks beneath beds (Seuront et al., 2019). The duration of aerial exposure caused by variation in vertical position within a mussel bed was also a strong determinant of heat wave survival (Benjamin et al., 2024). These observations highlight the importance of direct solar radiation on effects of temperature (Fig. 2) and diminish the sole use of air temperature as the critical environmental parameter setting the body temperature of intertidal organisms (Helmuth et al., 2006, 2002). The sole use of air temperature to estimate the impacts of AHWs on mass mortality can underestimate the predicted effect of the heat wave and overestimate physiological tolerance limits. A recommended approach would be to always use some metric of solar exposure in any models that attempt to predict heat wave impacts in intertidal zone ecosystems.

MHWs intense enough to cause mortality may generally be more damaging than AHWs for intertidal zone organisms. For example, in green-lipped mussels (*Perna canaliculus*), the number of hours exceeding 25°C while immersed was the strongest determinant of survival, despite the fact that the 6 h LT_50_ (see Glossary) in air was over 32°C (Sorte et al., 2018) and mean daily thermal maxima can be near 40°C (Benjamin et al., 2024). So, for those mussels, a persistent MHW of warm water is more damaging than dramatically higher temperatures experienced while in air. Though coral and kelp habitats are principally in the coastal subtidal zone, their vertical distribution limits abut the lowest parts of the intertidal zone, and their presence influences the ecology of intertidal zone ecosystems. Thus, we consider them in the context of how heat waves impact intertidal zone ecosystems. Strong mortality events associated with MHWs have been observed in coastal corals (Asner et al., 2022; Gardner et al., 2019; Riegl et al., 2018; Rubio-Portillo et al., 2016; Ruthrof et al., 2018; Shlesinger and van Woesik, 2023; Smale et al., 2019; Turicchia et al., 2018), threatening entire ecosystems that depend on their presence.

**Fig. 3.**
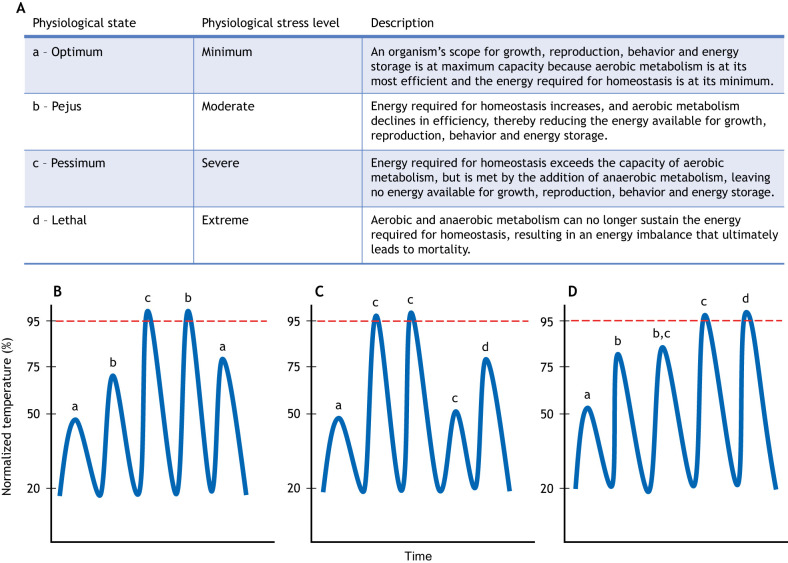
**Energetics plasticity in response to atmospheric heat waves in the intertidal zone.** (A) Definition of physiological states of energy-limited tolerance to stress, following Sokolova (2013). (B–D) Hypothetical temperatures and plasticity responses during successive low tide periods. Temperatures (*y*-axis) represent intertidal zone low-tide temperatures over five consecutive low tides at levels including below average (20th percentile), average (50th percentile), warm (75th percentile) and heat wave (95th percentile, red dashed line) temperatures (by definition, atmospheric heat waves are 2 consecutive days of 95th percentile temperatures). Different types of plasticity in response to those temperatures are indicated by physiological states (a, b, c, d from A) at each peak temperature in scenarios of plasticity including (B) heat hardening, (C) heat softening and (D) no plasticity. In the heat-hardening scenario (B), organisms lessen the impact of successive extreme heat exposure during the heat wave, and shift their optimal thermal range to warmer temperatures than prior to the heat wave exposure. In the heat-softening scenario (C), exposure to a heat wave causes increasing sensitivity to previously less-stressful temperatures, including lethal responses at temperatures that would not have been lethal prior to the heat wave exposure. In the no-plasticity scenario (D), cumulative effects of successive warm and heat wave days can cause increasingly severe physiological responses when there is no heat hardening.

Clearly, the duration of AHWs or MHWs is an important determinant of mortality outcomes. Heat waves with lesser maximum temperature intensity that persist for a long duration may cause as much or even more mortality than a brief heat wave that has a maximum temperature that far exceeds the critical thermal maximum (CT_max_; see Glossary). The basis for that is the prolonged detrimental effect of the heat waves on the regulation of physiological mechanisms and extension of energetic deficits that can result in higher rates of mortality (Sokolova, 2013, 2023) (Fig. 3). As the frequency and intensity of heat waves continue to increase, so too will mass mortality of intertidal zone organisms.

### Evolutionary adaptation

Individuals of sedentary intertidal zone tidepool organisms are sometimes also found in subtidal habitats. These individuals are known to be more thermally tolerant, reflecting selection and adaptation to the increased thermal extremes of the intertidal zone. Those differences can be observed among species [e.g. intertidal versus subtidal Tripterygiidae triplefin fish (Willis et al., 2021), and many other examples (Somero, 2002)] and in local adaptation of intertidal and subtidal populations of a single species. For example, local adaptation has been observed in *Porites lobata* corals that tolerate the extreme high temperatures in intertidal pools on the fringing reef of Ofu Island in American Samoa (Barshis et al., 2010). Those corals, which are a genetically distinct population from conspecifics living nearby on the cooler and more thermally stable forereef habitat, grew faster and maintained higher levels of heat shock proteins (see below) under common garden conditions (Barshis et al., 2018). A similar phenomenon has been observed in another species of *Porites* coral (*Porites lutea*) in tidepools in the intertidal zone of South China Sea shorelines (Huang et al., 2024). Those tidepool corals maintain a distinct physiological advantage under heat wave conditions by enhanced antioxidant capacity and strengthened maintenance of the symbioses that make up the full coral holobiont (Huang et al., 2024). Adaptation of tidepool corals to tolerate current and near-future increases in thermal extremes associated with heat waves is complicated to understand because of the taxonomic diversity of the coral holobiont (Quigley, 2024). Variation in the bleaching response to thermal extremes could be the product of selection for more heat-tolerant coral genotypes, but may also be due to selection of a more heat-tolerant endosymbiotic microbial and microalgal holobiont community (van Woesik et al., 2022). Exposure of coastal corals to heat waves consistently causes bleaching, but the magnitude of bleaching is inconsistent among locations, suggesting that local adaptation or other environmental factors also influence heat wave responses (Shlesinger and van Woesik, 2023). Significantly, there is some evidence that corals are adapting to extreme heat as temperatures that induce bleaching have increased during recent warming; however, this may only be an adaptive response in coral types that are generally stress resistant to begin with (i.e. *Porites* corals; McCarthy et al., 2024).

While the ‘classic’ mode of adaptation is natural selection acting on existing available genetic diversity (e.g. post-settlement mortality in corals from subtidal populations that settle in intertidal zone tidepools), there is growing evidence that heat waves can act to accelerate genetic diversification and thus facilitate stress-induced evolution (Love and Wagner, 2022; Mojica and Kültz, 2022). During heat waves, high temperatures elevate levels of mitochondrial flux and release of partially reduced oxygen, increasing levels of reactive oxygen species (ROS; see Glossary) (Freire et al., 2012). Oxidative stress resulting from increased production of ROS during exposure to extreme heat has been shown to produce adaptive mutations in populations of microbes (Sazykin and Sazykina, 2023). Extreme heat induces physiological changes as a consequence of homeostatic maintenance that increase genomic mutation rates by disruption of processes including DNA repair, shifting post-translational modification and altering transposable element activity, all with the potential to alter phenotypes and genotypes (Mojica and Kültz, 2022). Heat stress inhibits high-fidelity DNA repair methods including base and nucleotide excision and double-strand repair, causing cells to use low-fidelity methods (i.e. non-homologous end joining), increasing errors and causing mutagenesis (Mojica and Kültz, 2022). Heat stress induces heritable epigenetic processes including DNA methylation (see Glossary) and histone modifications, which modify chromatin and influence gene expression to alter phenotypes (Mojica and Kültz, 2022). Heat stress also inhibits transposable element silencing, which can cause shuffling of whole regions of chromosomes, reordering genes to produce evolutionary change (Heng and Heng, 2021). While such changes can occur in somatic tissues, they often cause deleterious consequences for already differentiated cells (e.g. cancer). However, changes such as those in the germline have the potential to produce offspring with thermal physiology phenotypes adapted to increased heat wave intensity that can persist across generations (Badyaev, 2005).

Initial adaptive responses following heat waves will likely thus occur as a form of transgenerational plasticity (TGP; see Glossary), in which environmental exposure of the parental generation causes shifts in phenotypic response to the same environmental exposure in offspring (Walsh et al., 2024). While mechanisms of TGP are well understood in placental mammals (Sze and Brunton, 2024) and birds (Ruuskanen, 2024), there have been only a few TGP studies in intertidal organisms, and those studies raise as many questions as they answer. In a study of maternally generated TGP in purple sea urchins (*Strongylocentrotus purpuratus*), simulated MHWs were shown to increase the size and heat tolerance of larvae from high temperature-exposed mothers (Chamorro et al., 2023). Additionally, the eggs from high temperature-exposed mothers had higher protein content, indicating that MHWs shifted the oogenesis process and egg quality (Chamorro et al., 2023). From that study, many questions remain, such as whether TGP would persist across multiple generations of *S. purpuratus*, what aspects of oocytes were altered and, as only one male was tested, whether different males would have resulted in the same TGP observations. Future TGP studies would benefit from examination of inter-individual variation across females within the population, as the study pooled oocytes from multiple females (Chamorro et al., 2023). In a study of paternally generated TGP in green lipped mussels, *P. cancliculus*, acclimation of fathers to simulated MHWs caused an increase of developmental abnormalities in larvae and an increase in thermal tolerance, but only when the larvae were also raised in warm conditions, indicating an interactive effect of paternal and offspring responses to temperature (Kozal et al., 2024). Veliger shell size was also found to be higher when larvae from MHW-exposed fathers were raised under MHW conditions (Kozal et al., 2024). While this study does demonstrate a paternal influence, the degree to which the paternal effect matters for the mussel population is unclear as the eggs were all from mothers only exposed to cooler conditions, which would not be the case in a natural heat wave.

In both studies (Chamorro et al., 2023; Kozal et al., 2024), the degree of TGP-induced change in thermal tolerance was small (0.1–0.3°C), suggesting that TGP would be an inadequate mechanism to increase offspring survival under heat wave conditions, when maximum temperatures increase by an order of magnitude larger than the increase in heat tolerance. The degree to which this level of plasticity reflects population-level responses to temperature cannot be determined from either study because of the pooling of gametes. Additionally, having both male and female parents exposed to the range of thermal conditions being investigated is important.

## Physiological plasticity of intertidal organisms in a heat wave context

### Heat hardening and the cellular stress response

Within-generation adjustments of physiology are necessary for organisms to persist during heat waves that disrupt homeostasis (Georgoulis et al., 2023). Heat hardening (see Glossary) is a mechanism commonly thought to help organisms ameliorate the impacts of subsequent exposure to extreme heat associated with heat waves (Zhang et al., 2021). Heat hardening occurs when one or more exposures to sublethal high temperatures induce or elevate heat-protective mechanisms. Those mechanisms can enhance heat tolerance levels and reduce the impact of extreme heat exposure (Fig. 3B). In some cases, only a single exposure to sublethal thermal stress causes heat hardening. California mussels, *Mytilus californianus*, and the limpet *Lottia digitalis* exposed to a single heat stress of 25°C to 35°C for 2 h exhibited greater thermal tolerance than conspecifics that did not receive a heat stress treatment (Pasparakis et al., 2016; Moyen et al., 2020). Sometimes, multiple consecutive heat exposures are necessary to induce heat hardening. For example, four exposures to 27°C induced heat hardening in the Mediterranean mussel, *Mytilus galloprovincialis* (Georgoulis et al., 2023). Heat hardening can also occur over longer time scales, such as seasonally, as a result of repeated exposure to sublethal temperatures during warm seasons. For example, summer acclimatization increased heat tolerance by 2–4°C in the razor clam *Sinonovacula constricta* (Zhang et al., 2021).

The cellular changes associated with heat hardening are complex and involve mechanisms for avoidance of protein misfolding, prevention of oxidative damage, regulation of cellular metabolism and cytoskeleton repair (Tomanek and Zuzow, 2010). Those changes support increased mitochondrial energy production while scavenging the resulting increased release of ROS and protecting macromolecular structures by the cellular stress response (CSR) (Kültz, 2005; Georgoulis et al., 2021). Intertidal zone mussels (*Mytilus trossulus* and *M. galloprovincialis*) exposed to extreme heat upregulated genes for cytoskeleton repair, suggesting thermal stress-induced damage to cytoskeletons (Tomanek, 2012). Heat hardened intertidal zone mussels (*Mytilus coruscus*) up-regulated pathways involving apoptosis, cell–cell adherens junctions and vasodilation, which together maintained integrity of the gill during heat stress (Dong et al., 2023). In the Mediterranean mussel, *M. galloprovincialis*, heat hardening involves mitochondrial electron transport system (ETS) enhancement by increasing expression of specific ETS proteins NADH dehydrogenase subunit 2 (ND2) and cytochrome *c* oxidase 1 (COX1), increasing phosphorylation of the energy balance-sensing protein AMP activated protein kinase (AMPK) (Georgoulis et al., 2021). Additionally, heat hardening of *M. galloprovincialis* is associated with increased cytosolic accumulation of specific free amino acids, potentially suggesting compatible solute system shifts associated with heat hardening (Georgoulis et al., 2023). Heat hardening is likely to require increased mitochondrial activities beneficial for producing the ATP needed for processes involved in the maintenance of homeostasis under increasing temperature (i.e. mitochondrial substrate oxidation rates).

A core feature of the CSR that likely influences heat hardening is the induction of heat shock proteins (HSPs; see Glossary) and other members of the cellular stress response (CSR) that protect macromolecular structures damaged by extreme heat (Kültz, 2005). HSPs are molecular chaperones that stabilize denaturing proteins during thermal stress and are ubiquitous across life (Tomanek, 2011; Tomanek and Zuzow, 2010). HSP70 is critical for setting thermal tolerance boundaries (Collins et al., 2023) and will often designate an organism's environmental niche (Dong et al., 2022). Because it is so influential to an organism's ability to undergo thermal stress, it often comes at a high cost to other energy-producing proteins, which can potentially impact growth and development (Tomanek and Sanford, 2003). The upregulation of HSPs during exposure to extreme heat is a core feature of the CSR in intertidal invertebrates (Tomanek and Sanford, 2003; Dong et al., 2008, 2022). For example, exposure of the intertidal limpet *Patella vulgata* to increasingly warm thermal extremes altered the expression of over 250 genes, the majority of which encoded HSPs (Moreira et al., 2021).

The intensity of CSR upregulation of HSPs in response to heat wave conditions is dependent on the thermal niche set by the organism's vertical zonation in the intertidal zone (Collins et al., 2023; Harada and Burton, 2019). Some intertidal invertebrates living at higher elevations maintain elevated levels of HSP70 in comparison to their lower elevation conspecifics, a preemptive heat-hardening strategy that provides a defense from unpredictable thermal stress exposure in thermally variable environments (Collins et al., 2023; Harada and Burton, 2019). Intertidal zone limpets from higher and lower intertidal zones maintained different levels of HSP70 expression: under ambient temperature conditions, higher intertidal limpets maintained higher HSP70 levels than low and mid-intertidal limpets (Dong et al., 2008). Heat stress induced the expression of HSP70 in low intertidal limpets at temperatures as low as 28°C, whereas the high intertidal zone limpets did not elevate HSP70 expression under any heat stress scenario (Dong et al., 2008). This implies that high intertidal limpets employ a preparative defense strategy with high levels of HSP70 in their cells as a mechanism for protection against extreme heat stress (Dong et al., 2008). Similar to the pattern of HSP70 levels and gene expression responses observed in limpets, low intertidal zone sculpins had a greater change in HSP70 when undergoing heat stress than sculpin species living higher in the intertidal zone, presumably due to the fact that they undergo heat stress less often than higher intertidal fish and must work harder to maintain homeostasis (Nakano and Iwama, 2002). Thus, how heat waves impact *hsp70* gene upregulation depends on the thermal thresholds of the heat wave as well as constitutive levels of the HSP70 protein present prior to the heat wave. In addition to the importance of vertical zonation in the CSR to heat waves, the nature of thermal shifts associated with the heat wave (i.e. gradual versus acute increase in thermal extremes) also plays an important role. The tidepool copepod *Tigriopus californicus* had stronger levels of *hsp70* expression during a gradual ramp up to the heat wave temperature of 36°C (1°C 20 min^−1^) as compared with an abrupt shift to 36°C for 1 h (Harada and Burton, 2019). Additionally, gradual thermal ramps led to greater expression of *hsp70* and *hspb1* than acute exposure (Harada and Burton, 2019). The authors speculated that gradual exposure to thermal stress allows enough time for HSPs to counteract protein denaturation while acute thermal stress is too sudden and extreme for HSPs to function effectively (Harada and Burton, 2019).

The ability of intertidal zone organisms to prevent oxidative damage of macromolecules by scavenging ROS is needed for heat hardening in order to safely maintain a higher mitochondrial flux induced by higher temperatures (Moreira et al., 2023). The CSR increases the activity of antioxidants that neutralize ROS (Istomina et al., 2013). Heat hardening in the intertidal zone mussel *M. galloprovincialis* follows increased expression of antioxidants including glutathione reductase, metallothionein, superoxide dismutase (SOD), glutathione *S*-transferase (GST) and catalase (CAT), indicating an increased ability to remove excessive ROS to prevent cellular damage (Feidantsis et al., 2020; Georgoulis et al., 2021). Acute heat shock in *M. galloprovincialis* and *M. trossulus* mussels led to increases in proteins related to the production of NADPH and the pentose phosphate pathway, likely as a strategy for producing ROS scavengers (Tomanek, 2011; Tomanek and Zuzow, 2010). A similar increase in antioxidant activity was observed in intertidal Manila clams, *Ruditapes philippinarum* (He et al., 2022a,b).

Heat hardening has also been shown to involve temperature compensation of overall metabolism by a reduction in mitochondrial activity and metabolic enzymes (Sokolova, 2023). Intertidal *R. philippinarum* experiencing AHWs (40°C) reduced the activity of many different ATPases, but maintained higher metabolic rates than at normal temperatures (He et al., 2022a,b). Heat-stressed *M. galloprovincialis* reduced levels of electron transport chain and Krebs cycle proteins (Tomanek, 2011). Intertidal rock pool goby fish, *Gobius paganellus*, decreased mitochondrial respiration and overall metabolism during heat wave conditions by reducing the activity of key enzymes including isocitrate dehydrogenase and lactate dehydrogenase (Paul et al., 2021).

### Heat softening and oxidative stress

Less commonly than heat hardening, moderate heat waves can reduce tolerance to subsequent exposure to extreme heat, a term we are coining ‘heat softening’ (Fig. 3C). In heat softening, intertidal zone organisms are more sensitive to even a small increase in temperature after having been exposed to one or more extremely hot periods of low tide during a heat wave. Cellular damage has been shown to occur during heat wave conditions as a result of thermal and oxidative stress. Proteins denature during thermal and oxidative stress which accompany extreme temperature exposure (Collins et al., 2023; Tomanek, 2012; Vinagre et al., 2012) and the over-abundance of ROS produced in cells exposed to heat wave conditions (Madeira et al., 2013; Vinagre et al., 2012), causing damage to lipids, proteins, DNA, free amino acids and carbohydrates (Madeira et al., 2013). Heat softening may be a consequence of the accumulation of damage faster than repair mechanisms can fix it (Sokolova, 2013).

Heat-softening responses are likely to involve thermal responses of mitochondrial function and energetics, as mitochondrial processes are central to organismal function (Sokolova, 2023). Mitochondrial processes are influenced by temperature (Blewett et al., 2022) and mitochondrial function may be constrained at thermal extremes, such as MHWs and AHWs (Chung and Schulte, 2020). Detrimentally, and especially at temperatures that may reduce optimal mitochondrial function (Chung and Schulte, 2020), those are the same processes that produce damaging ROS (Lushchak, 2010; Sokolova, 2013, 2023). In the intertidal limpet *P. vulgata*, extreme high temperatures repressed the activity of antioxidants, hinting that ROS removal might have been hampered by elevated temperatures characteristic of heat waves (Moreira et al., 2021). Heat softening was observed in blue mussels (*Mytilus edulis*) as a decrease in heat tolerance (LT_50_) of over 5°C during a 5 day period of successive daily exposures to a MHW (Seuront et al., 2019). Reduced expression of genes associated with oxidative stress, cellular apoptosis, energy production, circadian rhythm and a general stress response was observed following exposure of *M. edulis* to a MHW of +5°C for 20 days (Grimmelpont et al., 2024).

## Energetic consequences of physiological plasticity in response to heat waves

The above-described shifts in cellular physiology associated with heat wave exposure are energy intensive. Energetic shifts in response to heat waves that necessitate a greater proportion of an organism's available ATP to be shifted towards maintenance of homeostasis are almost certain to result in a reduction in the ATP available for other energetic processes including growth, reproduction, immune function and behavior (Sokolova, 2013).

### Reproductive consequences

The effects of heat wave exposure on reproduction have been widely observed in intertidal zone organisms. MHWs that stress reproductive adults may cause organisms to invest less energy into reproduction and therefore produce fewer, smaller, lower-quality gametes (Smith et al., 2023). Further, many intertidal organisms transmit gametes into the water column. With MHWs occurring more frequently, there is a higher chance of heat waves simultaneously occurring with external fertilization. Coinciding events could have positive or negative effects on fertilization and subsequent development (Kozal et al., 2024). Evidence for impacts of HW exposure on reproductive effort has been observed in intertidal zone organisms. For example, female *R. philippinarum* exposed to extreme MHW conditions (+10°C for 30 days) produced smaller oocytes, and downregulated vitellogenin and apolipoprotein proteins involved in yolk formation (Peruzza et al., 2023). Male *R. philippinarum* exposed to MHWs downregulated expression of genes involved in sperm motility flagella (Peruzza et al., 2023). Heat waves have also been shown to reduce fecundity and sperm production in *M. galloprovincialis* (Boni et al., 2016). Spawning rates under MHWs are significantly lower than under normal conditions (Shanks et al., 2020) for a number of reasons, including increased adult mortality, lack of efficient gametes (Lawrence and Soame, 2004) and the absence of ideal spawning conditions (Shanks et al., 2020). Decreased spawning points towards a fitness reduction in future generations and population decline. For example, the 2003 MHW near Venice lagoon likely caused a decrease in larval recruitment of *R. philippinarum* (Ponti et al., 2017). Other studies noted similar impacts in Pacific oysters (*Crassostrea gigas*) in Thau lagoon, France (Correia-Martins et al., 2022), as well as in Coos Bay Estuary, USA (Shanks et al., 2020).

MHW exposure has been shown to accelerate early development in sea stars (Balogh and Byrne, 2020) and cuttlefish (Coelho et al., 2022), but with differing impacts. Embryos of the direct-developing starfish *Parvulastra exigua* had faster development under MHW conditions, but fewer individuals survived development and those that did survive developed to a smaller body size (Balogh and Byrne, 2020), suggesting an energetic tradeoff. Additionally, >87% of survivors had body plans that deviated from the normal pentameric symmetry (Balogh and Byrne, 2020), suggesting that the higher temperatures disrupted body-plan regulatory mechanisms, including hox gene expression (Cisternas and Byrne, 2009). In contrast, intertidal embryos of the cuttlefish (*Sepia officinalis*) exposed to MHW conditions had a shorter development time and increased levels of SOD, suggesting elevated mitochondrial activity leading to increased production of ROS (Coelho et al., 2022). However, not all ROS deactivating enzymes assayed showed a similar pattern, as there was no MHW induction of CAT, GST or glutathione peroxidase, suggesting that the activity of SOD alone was enough to ameliorate the impacts of the MHW on ROS production, as no oxidative damage was observed to membrane lipids (Coelho et al., 2022).

### Behavioral consequences

Thermoregulatory behaviors, such as relocating to thermal refugia, occur prior to physiological adjustments. However, depending on the severity of the heat wave, behavioral responses may be impaired. A study on a marine gastropod, *Lunella granulata*, showed that during moderate MHWs, the organism quickly performed survival behavior, whereas during extreme MHWs, individuals were slower at performing the same responses, indicating they were impaired by more extreme temperatures (Joyce et al., 2022). MHW conditions have been shown to negatively impact burrowing capacity and filtration rates in the Manila clam (Peruzza et al., 2023). Reduced activity during and following thermal stress suggests a lasting performance detriment from exposure to temperatures above the thermal optimum (Joyce et al., 2024; Sokolova, 2013). As many intertidal organisms already live near their upper thermal limits (Smith et al., 2023), behavioral tactics such as looking for shade or digging deeper into sediment may require more energy and therefore increase their exposure to other stressors (Ishida et al., 2023). Similarly, extreme thermal stress can increase energy demand to upregulate physiological protective mechanisms, which requires higher metabolic energy and can then reduce the energy available for other behaviors (e.g. predator escape, reproduction and feeding) (Minuti et al., 2021). MHW conditions have been shown to have a larger impact on bold personality intertidal zone sea anemone individuals [i.e. those that engage in riskier foraging and exploration behaviors and as a result have higher metabolic rates (Maskrey et al., 2024)]. As high metabolic rates require a higher food intake, reduced capacity for feeding behavior due to thermal stress can cause a metabolic mismatch, resulting in impaired ecological performance and mortality (Minuti et al., 2021). Although metabolic rates decrease after returning to ambient temperatures, negative carry-over effects cause sustained physiological damage that prevents absolute recovery and can ultimately lead to mortality (Minuti et al., 2021). Potentially, then, heat wave conditions will act as a selective agent for lineages of individuals that have relatively inactive, or shy, personality traits associated with lower metabolic rates (Maskrey et al., 2024). Therefore, phenotypic adjustment may not lead to long-term resilience because of delayed biological responses and negative physiological carryover effects, indicating that MHWs and AHWs could have detrimental effects on intertidal ecosystems.

### Immune response consequences

Exposure to heat wave conditions has also been found to impact immune function. For example, *R. philippinarum* had higher frequencies of dead hemocytes following MHW exposure (Peruzza et al., 2023). In that study, the researchers also noted that MHW exposure decreased microfloral richness, with increases in pathogenic groups and decreases in beneficial groups (Peruzza et al., 2023). This shift suggests altered immune response and deviation towards dysregulated digestive processes, which could reduce the energy and essential nutrients assimilated from prey. The study also concluded that some physiological impacts from MHWs are likely sex specific. Male clams demonstrated significantly lower hepato-somatic indexes (the ratio of digestive tissue mass to total body mass) and higher antioxidant defense activity physiological changes that may reflect extra energy expenditure to protect their sperm from oxidative damage occurring during MHW conditions (Peruzza et al., 2023).

Heat waves directly reduce immune response and alter host–parasite relationships (Barber et al., 2016). For example, exposure to more than 1 week of heat wave conditions caused a reduction of hemocyte concentration and reduced immune system phenyloxidase activity in the snail *Lymnea stagnalis* (Leicht et al., 2013). Heat wave exposure increased the abundance of intertidal trematode parasite larvae in infected snails (Studer et al., 2010). Stickleback fish exposed to heat wave conditions (28°C, bordering on their upper thermal limit) became immunocompromised for around 2 weeks or longer after exposure (Dittmar et al., 2014). Observations of heat wave-induced fish die offs revealed mortality was highest in families with the highest average parasite loads (Wegner et al., 2008). Heat waves are therefore capable of increasing mortality risk by immunocompromising organisms and increasing parasite loads. In some cases, heat hardening can ameliorate the negative impacts of heat waves by boosting the immune system. For example, oysters acclimated to warm temperatures prior to simulated heat wave exposure showed improved immune responses (Scanes et al., 2023).

Not only is the immune function of intertidal zone organisms reduced during MHWs, but also bacteria, pathogens and opportunistic diseases are predicted to expand their range under increased temperature/climatic scenarios (Feehan and Scheibling, 2014; Harvell et al., 1999). *Vibrio* bacteria act as primary and opportunistic pathogens for marine invertebrates and are associated with increased rates of mortality (Zhang et al., 2023). MHWs provide an advantage to *Vibrio* bacteria as warmer temperatures accelerate bacterial growth and diversity, and energy reserves are routed away from immune function (Scanes et al., 2023; Zhang et al., 2023). Following a MHW in 2022, an increased abundance of *Vibrio* species was seen in corals that showed 90% or more tissue loss, lowering the relative abundance of important coral symbionts such as *Spirochaetacae* and *Endozoicomonas* (Prioux et al., 2023). Similarly, in organisms collected during a 2015 MHW, microbial communities shifted towards increased abundances of pathogenic bacteria (Rubio-Portillo et al., 2024). Therefore, MHWs increase the risk of mortality by increasing the growth and diversity of pathogenic bacteria while at the same time causing physiological stress, leading to a reduction in the immune response.

In addition to directly influencing immune response and pathogen abundance, MHWs are also capable of increasing susceptibility to environmental contaminants that also weaken immune defense. For example, MHWs increased the intake of the contaminant pharmaceutical carbamazepine (CBZ), which damaged hemocyte membranes in the intertidal zone mussel *M. galloprovincialis* (Nardi et al., 2022). The increased levels of CBZ occurred even after a period of recovery from the MHW and diminished the capacity of lysosomes to aid in the recovery of heat damage to membranes (Nardi et al., 2022). Additionally, co-exposure to CBZ and MHWs may damage acetylcholine-dependent neuronal signaling, which impairs the neuroendocrine immune system and can influence other processes in bivalves – such as phagocytosis – and is thus indicative of broader system damage beyond the immune system (Nardi et al., 2022). Oxidative stress associated with heat wave exposure may also exacerbate the neuroendocrine immune system impact of CBZ as a result of reduced functionality of hemocytes (Nardi et al., 2022).

## Future directions

Assessing the impacts of increasing frequency and severity of AHWs and MHWs on intertidal zone organisms and ecosystems remains an important scientific challenge. Combining classical approaches to monitoring organisms and populations within the intertidal zone ecosystems with modern approaches that leverage global-scale data from remote monitoring platforms has the capacity to transform how we assess when, where and the degree to which heat waves have impacted these ecosystems. In particular, the ease with which genomic-scale data can be generated from tissue samples facilitates assessment of selective sweeps at intertidal zone sites following heat waves. The use of increasingly high-resolution satellite data can aid in knowing when and where heat waves are likely to occur and facilitate research on the impacts of those heat waves on organismal, population and ecological levels of biological organization.

Determinants of physiological plasticity in response to heat wave conditions need to be better understood. For example, developing a stronger understanding of the interplay of heat-hardening and heat-softening mechanisms *sensu* Georgoulis et al. (2023) is of central importance for further studies. Other areas of future study could include the methylome, as paternally derived TGP would likely involve epigenetic changes. Future studies of TGP should also use a mating design that allows for individual variation in mothers and fathers to be assessed (e.g. a North Carolina Breeding design; Beavis et al. 2023). Ideally, the relevance of TGP as a type of adaptive evolution requires the persistence of changes through reproductive stages of the next generation, though we recognize the challenges involved in such studies, especially for organisms with planktonic larval stages. Some intertidal zone organisms possess life history traits that make such studies more tractable; for example, intertidal zone organisms that brood their offspring rather than releasing them to the plankton (e.g. *Leptasterias* sp. seastars), or have small body size and short generation time that facilitate rapid laboratory culture (e.g. *Tigriopus* sp. copepods), or are highly fecund or have pelagic larvae that are easily culturable such that a reasonable number of settlement-competent offspring can be achieved in laboratory conditions (e.g. many types of bivalve mollusks).

## Summary and conclusions

A consequence of continuing climate change is that AHWs and MHWs are likely to occur at increased frequency for longer durations at higher thermal maxima and over larger geographic areas. The intertidal zone ecosystem experiences both AHW and MHW conditions, and when those periods co-occur with maximal absorption of solar radiation due to the juxtaposition of low tide with solar midday, there can be dramatic impacts on intertidal zone communities. At the most extreme, the impacts are mass mortality. However, in most cases the impacts are likely to involve physiological shifts in energetics that will shift important traits such as growth, reproductive fitness, migration and species interactions, including response to pathogens. The ecological impacts of direct and indirect effects of AHWs and MHWs can involve shifting species distributions, and shifts in trophic cascades, which can restructure entire ecosystems when they involve keystone species. Those changes have a strong potential to impact the ecosystem services of intertidal zone communities, including for fisheries but also in wave attenuation and protection from sea level rise. The use of biomimetic data loggers has provided valuable long-term temperature records that can be used to develop novel remote-sensing methods in predicting intertidal zone thermal maxima, allowing for broader assessment of where, when and how much intertidal zones will be impacted by heat waves.

## Supplementary Material

10.1242/jexbio.246503_sup1 Supplementary information
