# Supplementary material for: Ecophysiological responses to heat waves in the marine intertidal zone
Source: J Exp Biol. 2025 Jan 16;228(2):JEB246503. doi: 10.1242/jeb.246503 (PMC11832128; doi:10.1242/jeb.246503)

Supplementary Materials and Methods

Combining Tide and Solar Radiation to Model Intertidal Temperatures

This document describes code that performs a new analysis to predict under-rock intertidal zone thermal maxima based solely on publicly available tidal predictions and solar radiation models.

This code is available at https://github.com/bbukaty/heat-wave-analysis.

To run the code, you will need Python 3 and a few scientific computing packages: `h5pyd, pandas, numpy, scipy, and matplotlib` .

```
In [ ]: import matplotlib.pyplot as
    plt import matplotlib.dates as
    mdates import scipy.stats as
    stats from utils import *
```

Data Sources

NOAA Tide Predictions

Info from NOAA here: https://tidesandcurrents.noaa.gov/PageHelp.html

This data source is an API where we can request current and historical tide predictions for sites along the California coast. We request predictions relative to the Mean Low Low Water level (MLLW); see `utils.py` for the full query parameters to this API.

NSRDB Physical Solar Model 3

Info from NSRDB here: https://nsrdb.nrel.gov/data-sets/us-data

> The National Solar Radiation Database (NSRDB) is a serially complete collection of hourly and half-hourly values of meteorological data and the three most common measurements of solar radiation: global horizontal, direct normal and diffuse horizontal irradiance. It covers the United States and a growing subset of international locations. These data have been collected at a sufficient number of locations and temporal and spatial scales to accurately represent regional solar radiation climates. For a given location covered by the dataset, it is possible to see the amount of solar energy that was at a given time, and to predict the potential future availability of solar energy based on past conditions.

Load NSRDB GHI Data

First, load some hardcoded information about the Fort Ross, CA site, where we have access to logged intertidal temperature data (see Gunderson et al. 2019).
See code 01_yearly_ghi.ipynb for how the closest NSRDB site was calculated; NOAA station ID was selected from their website manually.

```
In [ ]: site = SITES['Fort Ross']
        site
```

```
Out[ ]: {'name': 'Fort Ross',
         'tz': 'America/Los_Angeles',
         'loc': (38.51265, -123.24647),
         'noaa_station_id': 9416024,
         'nsrdb_site_id': 131123}
```

Next load the NSRDB GHI data for 2015 and 2016, corresponding to the years with the logged temperature data from Gunderson et al. 2019. Note that the NSRDB data were formatted with UTC timestamps, so the times were localized to Fort Ross's pacific time zone. `get_nsrdb_data` also caches data in `/cached` to reduce queries to the NSRDB API.

```
In [ ]: ghi_2015 = get_nsrdb_data(2015, 'ghi', site['nsrdb_site_id'], site['tz'])
        ghi_2016 = get_nsrdb_data(2016, 'ghi', site['nsrdb_site_id'],
        site['tz']) ghi_extended = pd.concat([ghi_2015, ghi_2016])
        ghi_extended.head()
```

```
Found cached/site-131123-data-2015.pkl, loading...
Found cached/site-131123-data-2016.pkl, loading...
```

```
Out[ ]: 2014-12-31 16:00:00-08:00    135 2014-12-
         31 16:30:00-08:00     57
         2014-12-31 17:00:00-08:00      0
         2014-12-31 17:30:00-08:00      0
         2014-12-31 18:00:00-08:00      0
         dtype: int16
```

Load Logged Temperature Data

The data includes two sets of temperature loggers, at high and low intertidal elevation. We used only the high elevation loggers in this analysis.

```
In [ ]: hot_rocks_df = get_hot_rocks_data()
        # apply boolean mask to get high elevation data, then select the temperature
        series year_temps_high = hot_rocks_df[hot_rocks_df['elev'] == 'high']['temp']
        year_temps_high.head()
```

```
Out[ ]: dt
         2015-06-19 00:02:00-07:00    11.0
         2015-06-19 00:04:00-07:00    11.0
         2015-06-19 00:05:00-07:00    10.5
         2015-06-19 00:05:00-07:00    11.0
         2015-06-19 00:07:00-07:00    11.0
         Name: temp, dtype: float64
```

Journal of Experimental Biology • Supplementary information

Get NOAA Tide Predictions via API

The requested time period includes one day of padding on each end; this is for the interpolation described below.

In [ ]:
```
both_years_padded = [get_year_padded(2015)[0],
get_year_padded(2016)[1]] print(both_years_padded) tide_series =
get_noaa_tide_preds(site, both_years_padded)
```

```
['20141231', '20170101']
```

Tide Interpolation

The data we get from NOAA will be daily predicted high and low tide values. To compare and combine the tide level and the higher resolution GHI data, we interpolate the water level between the high and low predicted tides with cubic interpolation.

In [ ]:
```
# Interpolate tide high/low data to get a tide value for every timestamp in
    the ghi tide_high_res = interpolate_tide_preds(tide_series,
    ghi_extended.index)

EXAMPLE_DATE = '2016-06-20' tide_series[EXAMPLE_DATE].plot()
tide_high_res[EXAMPLE_DATE].plot(title="Interpolated Tide Predictions, Visualized")
```

Out[ ]:
```
<Axes: title={'center': 'Interpolated Tide Predictions, Visualized'}, xlabel='t'>
```

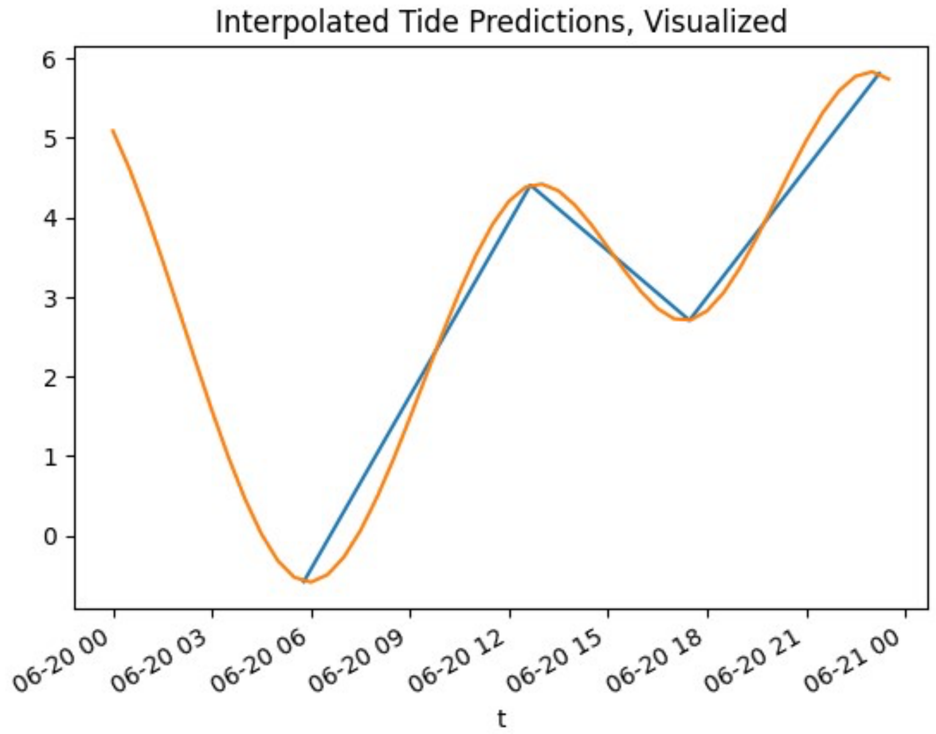

Journal of Experimental Biology • Supplementary information

Visualizing Data Sources

The below code creates a plot of all three data sources for one week in 2016. GHI is shown in red, predicted tide level in blue, and logged temperature in orange.

```
In [ ]: week_start, week_end = '2016-06-09', '2016-06-16'
         example_week_ghi = ghi_extended[week_start:week_end]
         example_week_temps = year_temps_high[week_start:week_end]
         example_week_tides = tide_high_res[week_start:week_end]
```

```
In [ ]:

         fig, ax1 = plt.subplots(figsize=(30, 4))

         # Plot GHI data
         ax1.plot(example_week_ghi.index, example_week_ghi, label='GHI',
         color='tab:red') ax1.xaxis.set_major_locator(mdates.DayLocator(tz=site['tz']))
         ax1.xaxis.set_major_formatter(mdates.DateFormatter('%Y-%m-%d', tz=site['tz']))
         plt.xlim(example_week_ghi.index[0], example_week_ghi.index[-1])
         ax1.set_ylabel('GHI', color='tab:red') ax1.tick_params(axis='y',
         labelcolor='tab:red')

         ax2 = ax1.twinx() ax2.plot(example_week_temps.index, example_week_temps,
         label='Low Tide Water Temp', ax2.tick_params(axis='y', labelcolor='tab:orange')

         ax3 = ax1.twinx() ax3.plot(example_week_tides.index, example_week_tides,
         label='Tide', color='tab:blu ax3.tick_params(axis='y', labelcolor='tab:blue')
         ax1.grid(True)

         fig.tight_layout()
         plt.show()
```

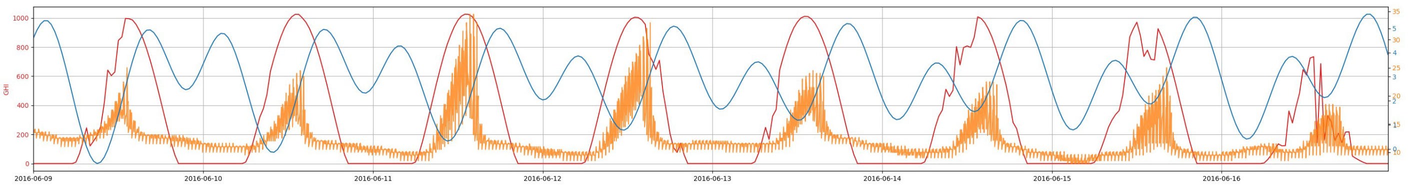

Journal of Experimental Biology • Supplementary information

Calculate Tide-Scaled GHI

To model the intuition that less solar radiation reaches the mid and low intertidal during periods of high tide, the next step is to scale the timestamped GHI data with the corresponding tide level.

First the tide data are normalized to a 0-1 range, where 0 is the lowest tide recorded over the period of logged temperatures and 1 is the highest. Then the tidal curve is inverted value by subtracting the normalized value from 1, so that the scaled tide is multiplied by 0 when the tide is high and 1 when the tide is low.

In [ ]:

```
# Normalize tide data to a 0-1 scale based on highest and lowest observed values
ov min_val, max_val = tide_high_res.min(), tide_high_res.max()
tide_values_normalized = (tide_high_res - min_val) / (max_val - min_val)

# Then invert the values so 1 is low tide, 0 is high tide
tide_values_inverted = 1 - tide_values_normalized

# Confirm we didn't mess up the data shape, before we try to
multiply assert tide_values_inverted.shape == ghi_extended.shape
tide_scaled_ghi = tide_values_inverted * ghi_extended
```

In [ ]:

```
example_week_scaled_ghi = tide_scaled_ghi[week_start:week_end]

fig, ax1 = plt.subplots(figsize=(30, 4))

ax1.plot(example_week_scaled_ghi.index, example_week_scaled_ghi, label='GHI',
color ax1.xaxis.set_major_locator(mdates.DayLocator(tz=site['tz']))
ax1.xaxis.set_major_formatter(mdates.DateFormatter('%Y-%m-%d', tz=site['tz']))
plt.xlim(example_week_scaled_ghi.index[0], example_week_scaled_ghi.index[-1])
ax1.set_ylabel('GHI', color='tab:red') ax1.tick_params(axis='y',
labelcolor='tab:red')

ax2 = ax1.twinx() ax2.plot(example_week_temps.index, example_week_temps,
label='Low Tide Water Temp', ax2.set_ylabel('Logged Temperature',
color='tab:red') ax2.tick_params(axis='y', labelcolor='tab:orange')
ax1.grid(True)

fig.tight_layout()
plt.show()
```

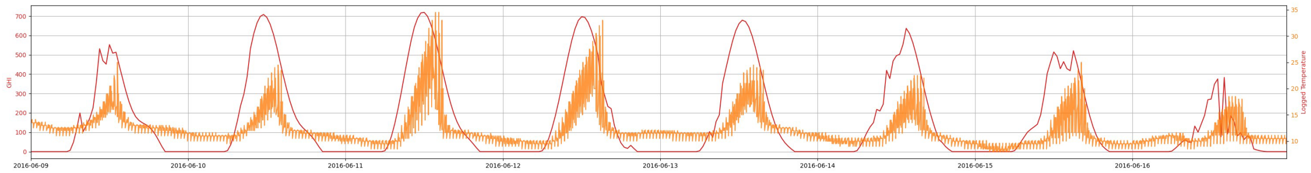

Journal of Experimental Biology • Supplementary information

Examine Model/Data Correlation

Next, the daily maximum tide-scaled GHI is determined and compared to the daily maximum recorded temperature using a regression analysis across the days of recorded data.

In [ ]:

```
# Resample logged temperature data and tide-scaled ghi data to daily maximum
values max_scaled_ghi = tide_scaled_ghi.resample('D').max() max_recorded_temp =
year_temps_high.resample('D').max()

# Confirm timezones are the same before we merge data into one
frame assert max_recorded_temp.index.tz == max_scaled_ghi.index.tz
df = pd.concat([max_scaled_ghi, max_recorded_temp], axis=1)
df.columns = ['max_scaled_ghi', 'max_recorded_temp']

# Drop rows where any of the values is NaN (this should only be days where
there's df = df.dropna()
```

In [ ]:

```
slope,        intercept,        r_value,        p_value,        std_err        =
stats.linregress(df['max_scaled_ghi']

print(f"Slope: {slope}")
print(f"Intercept: {intercept}")
print(f"R-squared: {r_value**2}")
print(f"P-value: {p_value}")
print(f"Standard error: {std_err}")
```

```
Slope: 0.0276
Intercept: 9.786
R-squared: 0.686
P-value: 8.292e-117
Standard error: 0.0008743
```

In [ ]:
```
# Map the colors to the entries in the frame colors
= df.index.month.map(lambda x: MONTH_COLORS[x])

# Create a scatter plot with specific month colors
plt.figure(figsize=(10, 6))
sc  =  plt.scatter(df['max_scaled_ghi'],  df['max_recorded_temp'],  color=colors,
alpha

# Add regression line x
= df['max_scaled_ghi'] y
= intercept + slope * x
plt.plot(x, y, color='black', label='Regression Line')  # Use a neutral color for
t

# Add invisible points for creating the legend for months
for month, color in MONTH_COLORS.items():
    plt.scatter([],      [],      color=color,      label=pd.to_datetime(month,
format='%m').month
plt.xlabel('Daily Maximum Tide-Scaled GHI') plt.ylabel('Daily Maximum

Recorded Temperature (℃)') plt.grid(True)

plt.legend(bbox_to_anchor=(1.05, 1), loc='upper left',

borderaxespad=0.) plt.show()
```

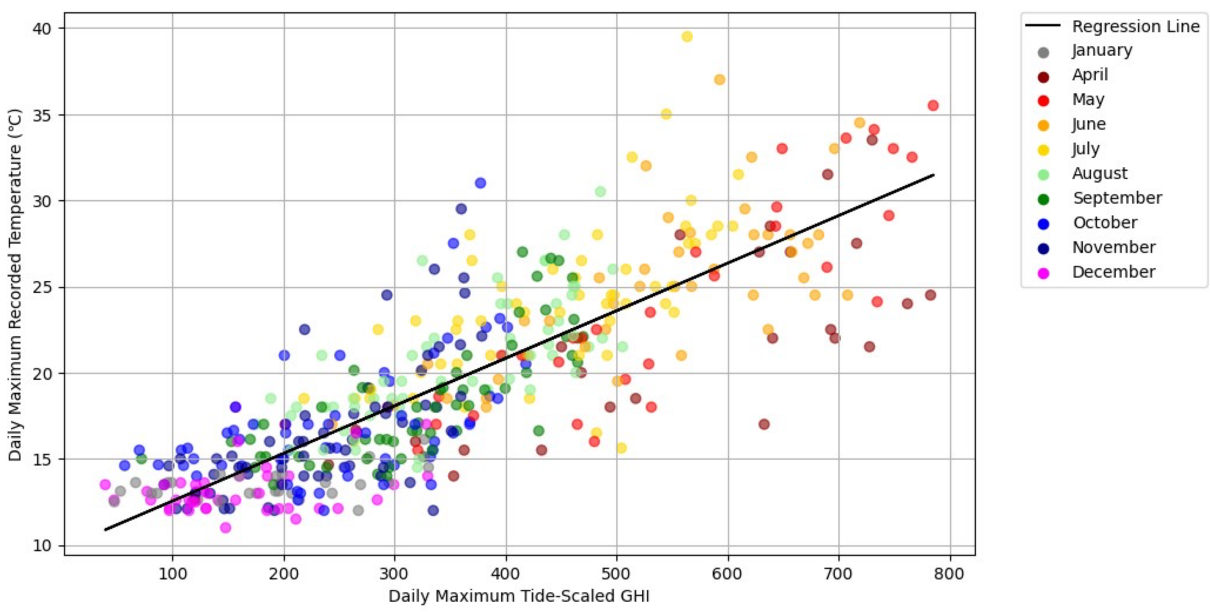

Supplement: Supplementary information [file jexbio-228-246503-s1.pdf]
